# Etiology of Milksickness: An Inaugural Thesis

**Published:** 1857-04

**Authors:** G. W. Wilkinson


### THE NORTH-WESTERN

### MEDICAL AND SURGICAL JOURNAL.

(NEW SERIES.)

VOL. VI. APRIL, 1857. NO. 4.

### ORIGINAL COMMUNICATIONS.

ARTICLE I.—*Etiology of Milksickness: an Inaugural Thesis for the Degree of M.D. in Rush Medical College, Session of* 1856–'7. By G. W. Wilkinson.

The reading in medical books and journals of the nature and cause of disease, and the remedies proper in their treatment, gives the student but a faint idea of the reality. And, as I am but a student, what I write must necessarily be theoretical, if not fanciful. And the subject I have chosen is the more difficult of writing upon from the fact, that the literature of the profession contains but little upon the subject, and that little is in the form of essays occasionally published in the medical journals of the day. In that isolated form it is not easy to arrive at any satisfactory conclusions. I will certainly not be expected to write anything very definite on a subject, on which Dr. Samuel Thompson represents the whole profession as being 'but inquirers and learners.'

Milksickness, as an endemic disease, is found in circumscribed localities through southern Indiana and Illinois, and it is also said to prevail in many parts of Kentucky and Alabama. The disease occurs almost uniformly in late autumn, and dry weather is especially favorable to its prevalence. We find the disease usually on low, marshy, or bottom-land, or the bluffs near by. Though such is generally the case, yet it is not uniformly so, but, as Dr. Byford remarks, 'their physical characteristics are not peculiar.'

We will now proceed to the consideration of that vexed question, the Etiology of Milksickness. There is great difference of opinion among writers on this subject, and the theories advanced are exceedingly numerous; but I believe there are but four of them deserving of particular notice. Those are, the Vegetable, Malarial, Animal, and Mineral. We will consider them in the order we have written them.

There is but little harmony of opinion among the advocates of the first theory, and they are not at all agreed as to what vegetable shall bear the blame of causing the disease.

Almost every person who has lived in a neighborhood where the disease prevails, and who believes in the vegetable theory, will say that he can point out the offending herb; but two of them can scarcely be found designating the same plant. In order that this theory look plausible, it would be necessary that the suspected vegetable be invariably found in localities where the disease prevails, and only there.

Dr. Drake is said to have advocated the vegetable theory; and if his Rhus Toxicodendron should be found where the disease prevails, and absent where it does not, then we would not dare deny that it was the cause. But such is not found to be the case.

A farmer on the high and dry land near Annapolis, Indiana, was in the habit of keeping a portion of his cattle over-night in a small lot, in which was no water and but little vegetation. Those cattle were attacked with sloes or milksickness, while the cattle on other portions of the farm were exempt. He excluded his cattle from the lot, and the disease never occurred on the farm afterward. Under the impression that the disease was of vegetable origin, he marked off the lot into narrow strips and went over the whole of it on his hands and knees, examining every spear of vegetation, but found nothing that did not grow equally abundant on other portions of the farm. In this case there could hardly have been a peculiar vegetable there and escape observation; therefore the disease was not of vegetable origin.

I will briefly add another case. In McLean County (this State), a farmer kept two separate lots of cattle in different enclosures, where was neither water nor vegetation. One lot of cattle had milksickness, while the others escaped. In this case the disease could not possibly have been caused by a vegetable product, for the cattle were all fed from the same corn, and watered from the same well.

This is probably sufficient consideration of the first theory, and we pass to the Malarial.

Were this disease of miasmatic origin, we should expect it to occur according to the following propositions:—

*First*—It would prevail in a direct ratio to the prevalence of the diseases that are by common consent called malarial.

*Second*—Wherever malarious diseases occur to any great extent, we should find milksickness.

We are perfectly familiar with the fact, that when heavy rains fall late in the Spring, causing inundations, which leave ponds and sloughs filled with water to stagnate under the heat of the summer sun, it causes whole districts to be so filled with the miasmatic influence that the inhabitants suffer excessively from intermittent and remittent fevers, and in their most grave form; but no one claims that milksickness occurred to greater extent in such cases. So far as I am informed, the past autumn has been attended with less than the usual malarious diseases, and more milksickness than has been before for many years. So we find that if milksickness prevails in a ratio to the miasmatic diseases, it must be in an inverse ratio.

We will now notice the second proposition:—

In Dr. Wood's *Practice*, we find the following language:—'Miasmatic diseases are apt to follow the submerging of meadows in order to increase their fertility, the forming of mill-ponds, and the damming of streams for the purposes of navigation. Neighborhoods, before remarkably exempt from disease, have thus become very unhealthy.' This is a fact too well known to admit of question, but no writer affirms that milksickness is ever so introduced along with the miasmatic diseases into neighborhoods where it had not prevailed before.

In western Illinois, we find the stagnant water, the decaying vegetation, and all the other requisites for the production of miasmata: and, just as we might expect, we find intermittent and remittent fevers prevailing to as great extent, and in as malignant form as in eastern Illinois; yet I am not informed of a single case of milksickness having been produced west of the Illinois River. Mackinaw Creek empties into the Illinois river a few miles below Pekin; and along the Creek to the very point where it empties into the River we find milksickness; while above and below along the River the disease never appears.

I had occasion the past autumn to go into that neighborhood, and stopped at a house near the Creek for dinner. Our landlady told us that we were in the midst of the milksickness, and that their cattle and those of their neighbors were dying of the sloes; but at the same time she assured us we need not be afraid to eat of the beef on the table, for it had been brought from Spring Lake. Now that Spring Lake is nothing but a slough of several miles in length, running almost parallel with the river, and is so marshy that cattle cannot cross it, though it is at some points but a few rods in width. This slough produces an abundant crop of vegetation, which falls down and decays on the mud, and presents the appearance of being the very hot-bed of miasmata; yet cattle graze about the sides of it and on the low bottom-land between it and the river with perfect impunity, and have never been known to have milksickness. But, at the same time, if we go a few miles up the River, to the mouth of Mackinaw, we find cattle dying by scores. Surely no one would contend that there was more malaria about a clear running stream as Mackinaw, than a stagnant swamp like Spring Lake.

This is all we propose writing on this point, and we pass now to consider some peculiarities which distinguish this from true malarious diseases.

*First, of the Odor*—This is described as something so peculiar as not soon to be forgotten when once observed. Practitioners who have treated this disease, say they are often able to form their diagnosis by the use of but one of their senses, and before entering the house. Dr. Byford has been endeavoring, by forming compounds, to produce an odor similar to that in this disease: and he thinks he has found the very article. Here is what he says of his discovery. 'It resembles almost exactly a strong odor of chloroform mixed with animal secretions. Say a smell of chloroform, and the breath of a patient salivated.' Whether or not this is a just comparison, it is not my purpose to decide. That there is an odor accompanying this disease, which cannot be compared nor mistaken for that of any other, is sufficient for my purpose.

Another characteristic is that one attack powerfully predisposes to subsequent ones which may be induced by over-exertion, even though the person should go to a place distant from where the disease prevails. An instance of this peculiarity I propose giving in a subsequent part of this paper.

Next in order is the Animal Theory, which will engage our attention for a time.

This theory is advocated by a few as the only source, and acknowledged by most writers to be one of the sources, if not the only one, of this disease. It has been observed that dogs, after feeding on the flesh of a beast dead of a peculiar disease, are attacked with a disease in every respect analagous to that of which the beast died; and in both instances the disease is called sloes, trembles, milksickness, &c. From this fact we would very naturally infer that the disease in the beast had poisoned the flesh to such a degree that the dog had received it with the ingesta. But Dr. S. W. Thompson would have us think otherwise: and in his Essay, published in the *N.-W. Journal* for Sept. 1854, he writes thus on this point:—'Those attacked are not always the ones taking the largest amount of diseased flesh, but rather such as stay longest in close proximity to the noxious effluvia arising from the carcass: and as the beast usually dies in or close to the locality where the disease is supposed to originate, it is but fair to admit that the dog was subject to the same cause of disease as the cow. And, again, if there is any peculiar essential poison in the dead cow, we should reasonably expect to see the usual manifestation of the poison in the animal partaking thereof, and these effects should be most plainly exhibited in such as had taken the largest amount of matter containing it.' He answers his own objection very well in these words:—'It is true that some men (and this fact applies equally to other animals) are more susceptible of a given amount of some agents than others, and this may possibly be the case in the present instance.' We consider this not only possibly the case, but extremely probable, especially so when we consider some other facts in connection. Farmers, living in neighborhoods where milksickness prevails, are well aware, that to prevent their dogs dying of the disease it is only necessary that they be so confined that they get none of the diseased meat. I have seen dogs confined within a few rods of a cow dead of milksickness, and was informed that they had learned from experience that their dogs so confined were in no danger whatever of having the disease. Therefore I think it an established fact that dogs do receive the disease from meat, and seldom, if ever, from other sources. The disease is not only communicated to dogs in this manner, but man may also receive it from diseased meat. To prove this point, I will report a case as I received it from one of the suffering party.

Mr. D. C. Wood and family resided in Greenfield, Indiana, and as milksickness had never appeared in their immediate neighborhood they had no fears of it, and observed none of those precautions which are usually observed where the disease prevails. One day in autumn, a man sold beef in the village, and one or more of each family that ate of it were attacked with milksickness. Some of those attacked died, and others recovered, if a person can properly be said to recover who survives. When those persons were simultaneously attacked with similar symptoms, they were satisfied the beef had been the cause, and so they traced it to its origin, and found it had been brought eighteen miles, and from a neighborhood of milksickness where cattle were dying of the disease.

Mrs. Wood's case presents an instance of that peculiarity of the disease already noticed, and which I promised to refer to again. She now lives in Peoria, Illinois, and is still subject to the disease whenever she greatly fatigues herself by over-exertion, and each attack presents all the symptoms of the original one, not even excepting the peculiar odor: and this, too, in a place where the disease is acknowledged to never occur as an endemic.

Instances of the above kind are so very common, that the popular opinion that the flesh of animals affected with the disease is capable of producing it in man, is amply sustained by facts. And not only the flesh, but the milk and butter may produce the same effect.

Cases are reported where families had abstained from beef, milk, butter, etc. and still were not exempt from the disease. Such cases do not prove that the disease is never received from animals; but only argues that it may be derived from another source: and that such is the case I am ready to acknowledge. For, when we have traced the disease to the animal, we have not yet found the prime cause, as whatever causes it in the cow, may, under some circumstances, cause it in man.

This brings us to the consideration of the fourth and last of the theories.

We are convinced in our own mind that the prime cause of the disease is a mineral substance contained in the earth, at or near the surface, in certain localities; and being taken into the system of man or beast produces that series of symptoms constituting the disease called milksickness. We are not prepared, as we would wish to be, with an array of facts derived from observation and experiment, to show what that substance is; therefore we will have to depend for the proof of our theory wholly upon deductions drawn from the facts that have come to our knowledge. We think it would not be claiming too much to suppose the substance to exude from the earth, and, under favorable circumstances, accumulate on the surface in such quantity as to be taken into the system of the cow along with the grass, corn, or salt, and produce the disease.

What are the circumstances favorable to the accumulation on the surface? Evidently the very dry weather of late Autumn, when there are no rains to wash it away. When frost has killed the greater part of the vegetation, cattle are obliged to graze near the surface to gain sustenance, and thus are more liable to get the poison with their food: and at this season of the year the disease prevails to the greatest extent, and continues till wet weather begins.

We think this theory will account for the appearance of the disease in all cases where we have known it to occur. On the farm near Annapolis, Indiana, we may readily suppose that the cattle in the lot had taken the poison with their salt or food; and the reason that the cattle did not contract the disease on other portions of the farm, was, that the poison was not at the surface in any other place. In the case in McLean County, Illinois, the substance was probably at the surface in one lot, and not in the other.

We are disposed to think that the earth through the whole southern part of Indiana and Illinois contains this substance, though it is not at the surface in all places. We incline to this opinion from the fact that the disease in some neighborhoods appears to be caused entirely by the water of a spring. The mineral in those springs appears to be in too dilute state to cause the disease until the surface water is dried up.

A student of the present class in the College says, that the farmers in some portions of the southern part of this State notice that when their cattle, for want of water in other places, are obliged to resort to certain springs, usually contract the disease; and if kept from those springs they escape. A case of this kind came under our own observation, where the enclosing of the spring, so that cattle could not get to it, was followed by the disappearance of the disease from the neighborhood.

This is all we had proposed writing, and is probably more than we should have written unless we could write more to the point; but, since writing the above, we have noticed an article in the *Bloomington Pantagraph*, taken from the *Edwardsville Advocate*, and headed 'The Milksick:' and notwithstanding the fact, that newspaper articles have by some means fallen into disrepute, and so far as we know are not taken as good authority on medical subjects; yet we will venture to append an extract, which of course we will not consider you bound to receive as orthodox.

'A few days since, while walking over what is familiarly known as Tan-Yard Hill, a place notorious for the milksick, we observed that the earth was spotted with a white mold, not very thick nor in very large quantities—each spot being at the most not larger than a dime, and frequently several feet from any other. It appeared to have exuded from the earth, and pressed between the fingers had more the substantial properties of a mineral than a mold. It at once occurred to us that this very article might be the milksick. It may possess saline qualities in composition with arsenic, and hence prove a temptation to cattle.

'In mentioning the existence of this mold or mineral poison to a farmer, well known in these parts, we were informed by him that he had observed his cattle lick it up when they had access to it, and in no instance without taking the milksickness, and in most cases fatally.

'That the three-leaved poisoned oak is not the milksick, we think is fairly inferable from the fact that it exists in almost all regions where the name of milksickness is unknown, and that many fields where it does not exist, are absolute milksick graveyards.

'In fact it is impossible that this terrible scourge should arise from any common well-known vegetable. The experience of thirty, forty, or fifty years, would have surely detected and exterminated any poison above ground. It must come from beneath the surface of the earth.'

We have copied the above article for want of better authority, and it may be taken for just what it is thought to be worth.

From what we have written it will be seen, that we believe milksickness to be a disease *sui generis*, and that it arises from a peculiar poison which man generally receives through the animal creation, but not invariably so. We believe it possible for man and ox to drink of the same water—the ox take the disease and man escape; from the fact that the ox drinks more according to size than man: but man may get enough of the poison to affect him, and thus receive it primarily.

*January*, 1857.

